# Chemometric Profile of Root Extracts of *Rhodiola imbricata* Edgew. with Hyphenated Gas Chromatography Mass Spectrometric Technique

**DOI:** 10.1371/journal.pone.0052797

**Published:** 2013-01-10

**Authors:** Amol B. Tayade, Priyanka Dhar, Jatinder Kumar, Manu Sharma, Rajinder S. Chauhan, Om P. Chaurasia, Ravi B. Srivastava

**Affiliations:** 1 Defence Institute of High Altitude Research, Defence Research & Development Organisation, Leh-Ladakh, Jammu & Kashmir, India; 2 Department of Pharmacy, Jaypee University of Information Technology, Waknaghat, Solan, India; 3 Department of Biotechnology & Bioinformatics, Jaypee University of Information Technology, Waknaghat, Solan, India; University of Sassari, Italy

## Abstract

*Rhodiola imbricata* Edgew. (Rose root or Arctic root or Golden root or Shrolo), belonging to the family Crassulaceae, is an important food crop and medicinal plant in the Indian trans-Himalayan cold desert. Chemometric profile of the n-hexane, chloroform, dichloroethane, ethyl acetate, methanol, and 60% ethanol root extracts of *R. imbricata* were performed by hyphenated gas chromatography mass spectrometry (GC/MS) technique. GC/MS analysis was carried out using Thermo Finnigan PolarisQ Ion Trap GC/MS MS system comprising of an AS2000 liquid autosampler. Interpretation on mass spectrum of GC/MS was done using the NIST/EPA/NIH Mass Spectral Database, with NIST MS search program v.2.0g. Chemometric profile of root extracts revealed the presence of 63 phyto-chemotypes, among them, 1-pentacosanol; stigmast-5-en-3-ol, (3β,24S); 1-teracosanol; 1-henteracontanol; 17-pentatriacontene; 13-tetradecen-1-ol acetate; methyl tri-butyl ammonium chloride; bis(2-ethylhexyl) phthalate; 7,8-dimethylbenzocyclooctene; ethyl linoleate; 3-methoxy-5-methylphenol; hexadecanoic acid; camphor; 1,3-dimethoxybenzene; thujone; 1,3-benzenediol, 5-pentadecyl; benzenemethanol, 3-hydroxy, 5-methoxy; cholest-4-ene-3,6-dione; dodecanoic acid, 3-hydroxy; octadecane, 1-chloro; ethanone, 1-(4-hydroxyphenyl); α-tocopherol; ascaridole; campesterol; 1-dotriacontane; heptadecane, 9-hexyl were found to be present in major amount. Eventually, in the present study we have found phytosterols, terpenoids, fatty acids, fatty acid esters, alkyl halides, phenols, alcohols, ethers, alkanes, and alkenes as the major group of phyto-chemotypes in the different root extracts of *R. imbricata*. All these compounds identified by GC/MS analysis were further investigated for their biological activities and it was found that they possess a diverse range of positive pharmacological actions. In future, isolation of individual phyto-chemotypes and subjecting them to biological activity will definitely prove fruitful results in designing a novel drug.

## Introduction

To identify and evaluate the therapeutic potential of medicinal herbs, isolation of active components and structural elucidation of these compounds is very essential in medicinal chemistry and natural product research. In recent years a lot of attention has been given towards the study of organic compounds from medicinal herbs and to elucidate their pharmacological activities. Numerous extraction techniques and analytical systems like spectrophotometry, capillary electrophoresis, high performance liquid chromatography (HPLC), high performance thin layer chromatography (HPTLC), gas chromatography (GC) with flame ionization detection (FID), gas chromatography/mass spectrometry (GC/MS) have been developed for the analysis and characterization of active compounds from medicinal plants. GC/MS has become an ideal technique for qualitative and quantitative analysis of volatile and semi-volatile compounds of plant origin. It has the unique combination of a perfect separation system (GC) with the excellent identification and confirmation technique (MS) which has made it the best suited analytical system for plant compound characterization. Additionally, for rapid extraction and precise analysis of these active phyto-compounds, the experimental design should also be optimized to obtain enhanced recoveries, low solvent consumption, and reduced extraction time [1]–[5].

*Rhodiola imbricata* Edgew. (Rose root/Arctic root/Golden root/Shrolo), belonging to the family Crassulaceae, is an important food crop and medicinal plant in the high altitude region of Indian trans-Himalayan cold desert. It is a popular medicinal plant in Pakistan, Nepal, India, Tibet, China, and many other countries and is widely used as food and traditional medicine around the world. A number of metabolites like phenylpropanoids, phenylethanol derivatives, flavanoids, terpenoids, and phenolic acids have been found in good quantity from these *Rhodiola* species and extracts of these plant species, particularly those from roots, have been shown to possess pharmacological activities. A survey of the literature showed that *Rhodiola* species influence a number of physiological functions including neurotransmitter levels, central nervous system activity, and cardiovascular function. It is being used to stimulate the nervous system, decrease depression, enhance work performance, eliminate fatigue, and prevents high-altitude sickness. Most of these effects have been ascribed to constituents such as salidrosides (rhodiolosides), rosavins, and p-tyrosol. Many pharmacological studies on *R. imbricata* have demonstrated that this plant exhibits cardioprotective, anti-inflammatory, antistress, dermal wound healing, and adaptogenic activities. It has also been found to possess antioxidant, antiaging, immuno-stimulant, radioprotective, and anticarcinogenic properties [6]–[24]. All these reports validate its use in traditional system of medicine.

However, the phytochemistry of the most important plant part having the medicinal and therapeutic potential, the root of *R. imbricata* has not been studied in considerable details. Hence, aim of the present investigation was to identify and quantify the chemotypes extracted successively in different solvents such as n-hexane, chloroform, dichloroethane, ethyl acetate, methanol, and 60% ethanol, from roots of *R. imbricata* from trans-Himalayan cold desert of Ladakh, India, by hyphenated GC/MS technique.

## Materials and Methods

### Chemicals

n-Hexane, chloroform, dichloroethane, ethyl acetate, methanol, ethanol, and water CHROMASOLV HPLC grade and all other chemicals used were of analytical grade and purchased from Sigma-Aldrich (St. Louis, MO, USA).

### Ethics statement

All necessary permits were obtained for the described field studies. The permit was issued by Dr. B. Balaji (IFS), Divisional Forest Officer, Leh Forest Division, Jammu & Kashmir, India.

### Plant materials and extraction

*R. imbricata* roots were collected from the trans-Himalayan region (Chang-La Top, altitude = 5330 m above mean sea level, Indus valley, Ladakh) of India in the month of October, 2011 after the period of senescence, with the prior permission from the local authorities. The plant roots were washed thoroughly and cut into small pieces and shade dried at room temperature for 15 days. Then they were finely powdered and used for extraction. The root powder (20 gm) was taken for the sequential extraction in six solvent systems with increasing polarity *viz.* n-hexane, chloroform, dichloroethane, ethyl acetate, methanol, and 60% ethanol by Soxhlet apparatus (Borosil GlassWorks Limited, Worli, Mumbai, India) at 40°C. The extracted fractions were concentrated under vacuum and reduced pressure (BUCHI Rotavapor R-205, BUCHI Labortechnik AG CH-9230, Flawil, Switzerland) at 40°C by circulation of cold water using thermostat maintained at 4°C in order to minimize the degradation of thermolabile compounds. The dry extracts were then stored in a −80°C freezer till further analysis.

### Preparation of sample for GC/MS analysis

The 25 mg of concentrated n-hexane, chloroform, dichloroethane, ethyl acetate, methanol, and 60% ethanol root extracts were redissolved in the respective solvents, vortexed properly and filtered through 0.22 µm syringe filter (Millipore Corp., Bedford, MA, USA). One microlitre aliquot of the sample solution was injected into the GC/MS MS system for the requisite analysis.

### Instrumentation and chromatographic conditions

GC/MS analysis was carried out on a Thermo Finnigan PolarisQ Ion Trap GC/MS MS system comprising of an AS2000 liquid autosampler (Thermo Finnigan, Thermo Electron Corporation, Austin, TX, USA). The gas chromatograph was interfaced to a mass spectrometer instrument employing the following conditions *viz.* Durabond DB-5 ms column (30 m×0.25 mm×0.25 µm), operating in electron impact [electron ionisation positive (EI^+^)] mode at 70 eV, helium (99.999%) was used as carrier gas at a constant flow of 1 ml/min, an injection volume of 0.5 EI was employed (split ratio of 10∶1), injector temperature 280°C, and transfer line temperature 300°C. The oven temperature was programmed from 50°C (isothermal for 2 min), with gradual increase in steps of 10°C/min, to 300°C. Mass spectra were taken at 70 eV, a scan interval of 0.5 s, and full mass scan range from 25 m/z to 1000 m/z. The data acquisition was performed on Finnigan Xcalibur data acquisition and processing software version 2.0 (ThermoQuest, LC and LC/MS Division, San Jose, California, USA).

### Identification of components

Interpretation of mass spectrum of GC/MS was done using the NIST/EPA/NIH Mass Spectral Database (NIST11), with NIST MS search program v.2.0g [National Institute Standard and Technology (NIST), Scientific Instrument services, Inc., NJ, USA]. The mass spectrum of the unknown component was compared with the spectrum of the known components stored in the NIST library. The name, molecular weight, and structure of the components of the test materials were ascertained.

## Results

**Figure 1:**
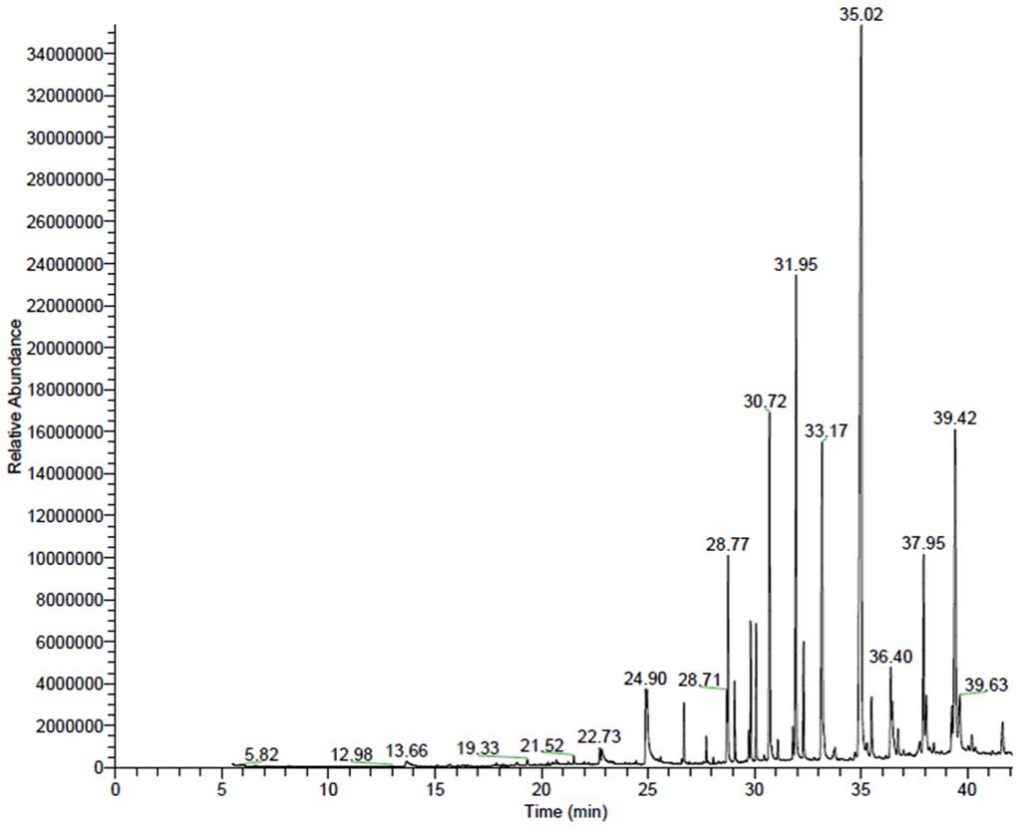
GC/MS chromatogram of n-hexane root extract of *R. imbricata*.

**Figure 2:**
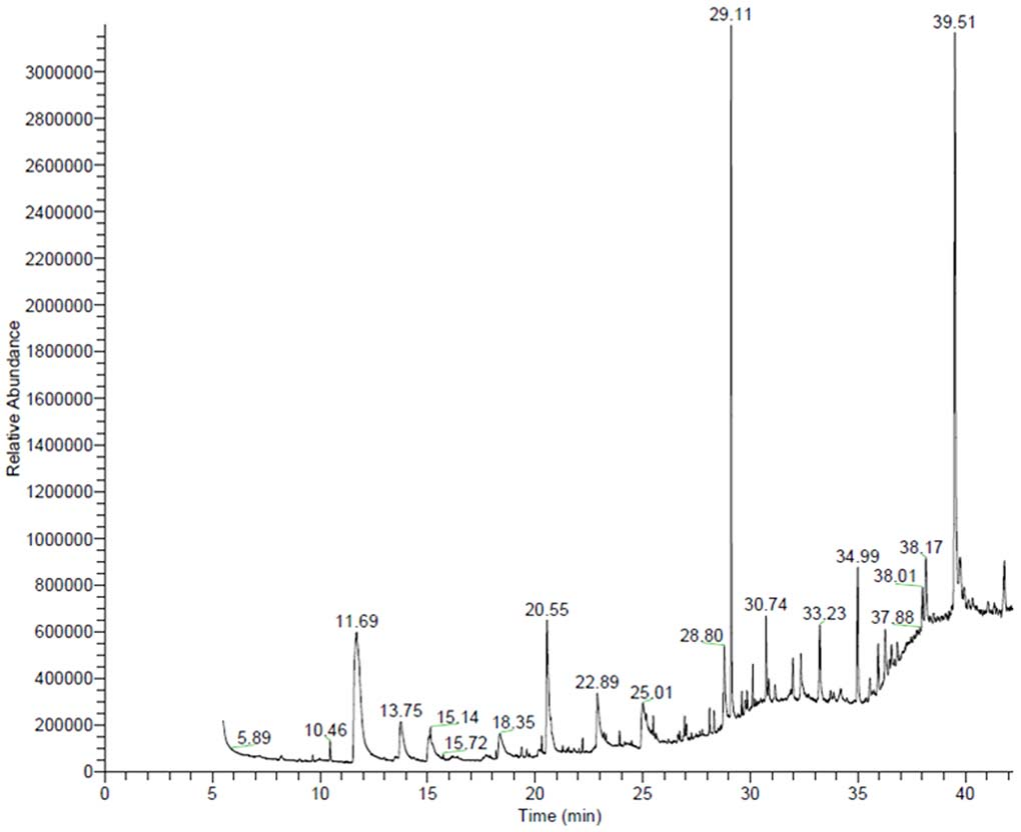
GC/MS chromatogram of chloroform root extract of *R. imbricata*.

**Figure 3:**
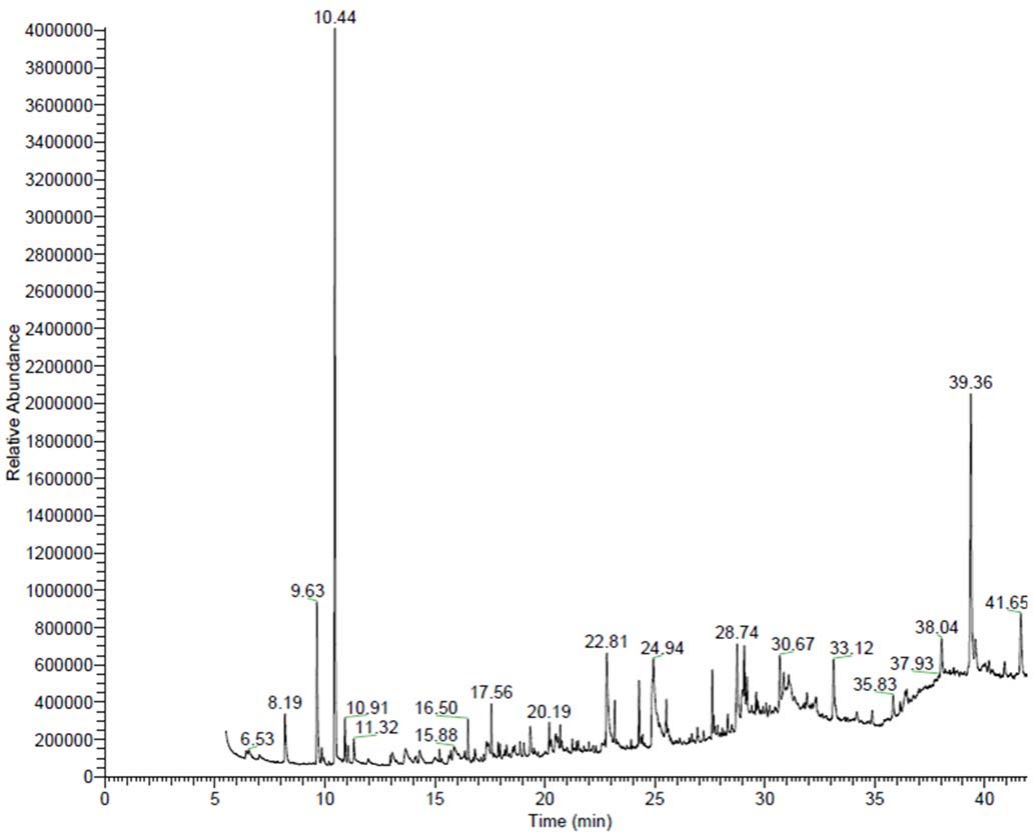
GC/MS chromatogram of dichloroethane root extract of *R. imbricata*.

**Figure 4:**
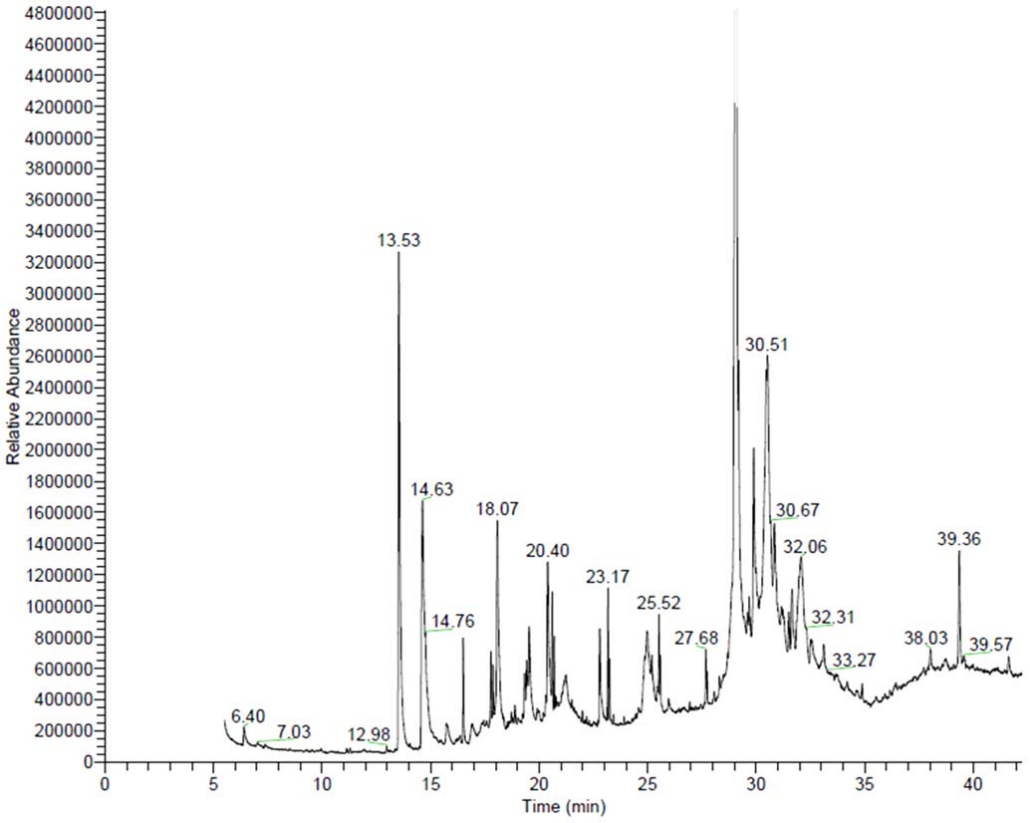
GC/MS chromatogram of ethyl acetate root extract of *R. imbricata*.

**Figure 5:**
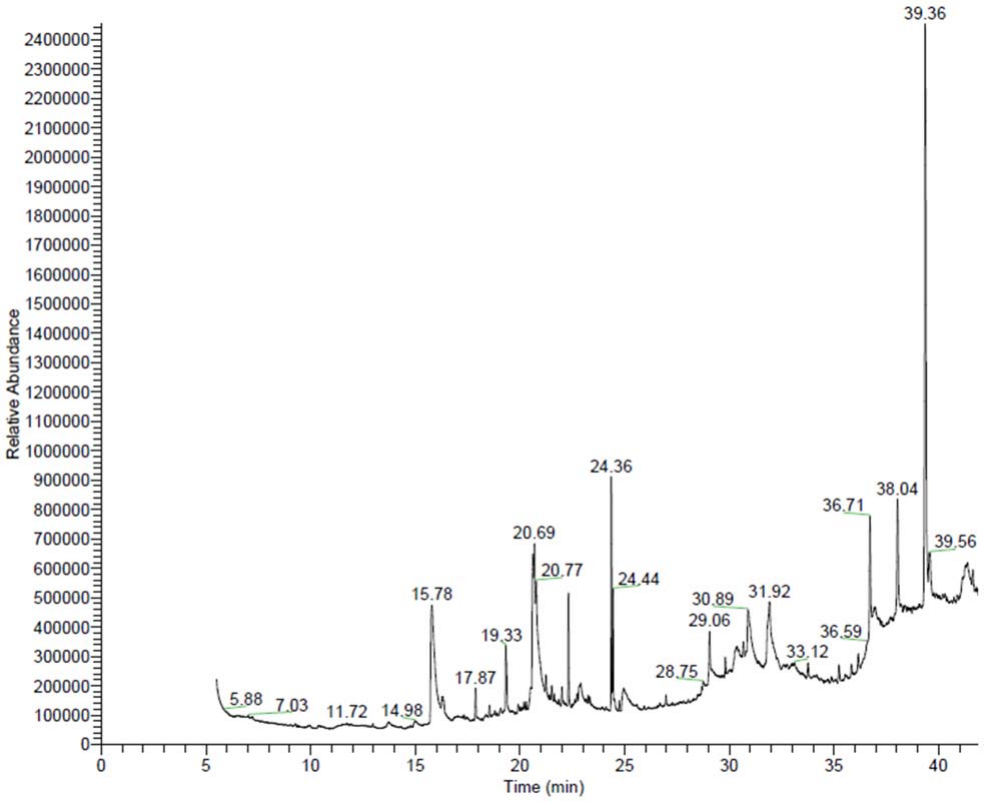
GC/MS chromatogram of methanol root extract of *R. imbricata*.

**Figure 6:**
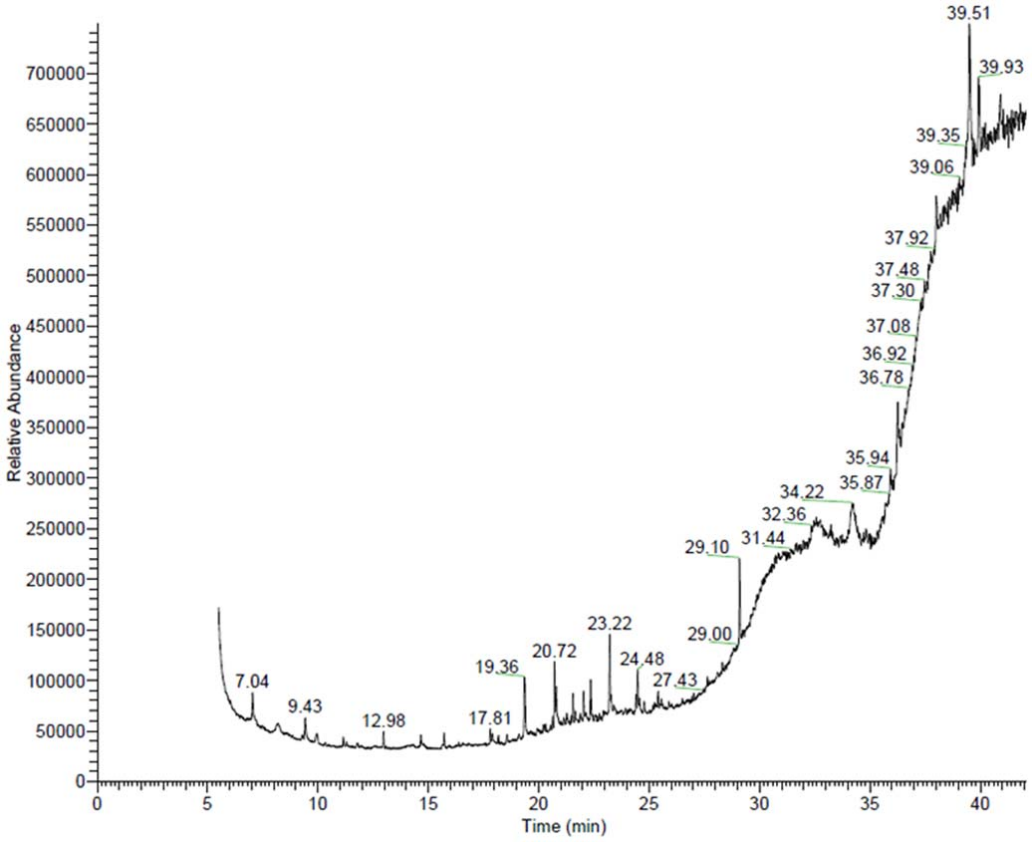
GC/MS chromatogram of 60% ethanol root extract of *R. imbricata*.

GC/MS chromatograms of n-hexane (Fig. 1), chloroform (Fig. 2), dichloroethane (Fig. 3), ethyl acetate (Fig. 4), methanol (Fig. 5), and 60% ethanol (Fig. 6) root extracts of *R. imbricate* as per the experimental procedure discussed above, showed various peaks indicating the presence of different chemotypes in the respective extracts.

### GC/MS chemometric profile

#### n-Hexane root extract

The n-hexane root extract revealed the presence of 22 different chemotypes which were characterized and identified (Table 1, Fig. 1) by comparison of their mass fragmentation patterns with the similar in NIST database library. Of these 22 chemotypes, 1-pentacosanol (28.21%), stigmast-5-en-3-ol, (3β,24S) (13.40%), 1-teracosanol (9.23%), 1-henteracontanol (8.53%), 17-pentatriacontene (7.01%), and 13-tetradecen-1-ol acetate (6.40%) were found to be major constituents whereas 1-hentriacontane (3.66%), 1-heptacosane (3.47%), 1-tericosanol (2.51%), 13-docosan-1-ol, (Z) (2.12%), eicosen-1-ol, cis-9 (1.99%) 1,30-triacontanediol (1.49%), stigmast-4-en-3-one (1.28%), bis(2-ethylhexyl) phthalate (1.20%), hexadecanoic acid (1.16%), 1-tetrateracontane (0.90%), campesterol (0.90%), α-Tocopherol-β-D-mannoside (0.73%), stigmastanol (0.71%), 1-pentatriacontane (0.48%), and 3-methoxy-5-methylphenol (0.46%) were found to be present in trace amount.

**Table 1:** Phyto-chemotypes identified in the n-hexane root extract of *R. imbricata* by GC/MS.

S. No.	Peak RT (min)	Peak area	Peak area (%)	Compound detected	Hit	SI	RSI	Prob	CAS No	Mol. Formula	Mol. Wt.
1	13.66	3489208	0.46	3-Methoxy-5-methylphenol	1	838	874	72.98	3209-13-0	C_8_H_10_O_2_	138
2	22.84	9266063	1.16	Hexadecanoic acid	1	801	842	73.13	57-10-3	C_16_H_32_O_2_	256
3	24.91	34516141	4.16	Ethyl linoleate	1	839	876	17.81	544-35-4	C_20_H_36_O_2_	308
4	26.69	6791237	0.9	1-Tetratetracontane	1	827	829	10.27	7098-22-8	C_44_H_90_	618
5	27.74	3769798	0.48	1-Pentatriacontane	1	823	838	11.01	630-07-9	C_35_H_72_	492
6	28.77	30375043	3.66	1-Hentriacontane	1	848	855	14.33	630-04-6	C_31_H_64_	436
7	29.07	9600422	1.2	Bis(2-ethylhexyl) phthalate	3	855	866	18.65	117-81-7	C_24_H_38_O_4_	390
8	29.82	20564432	2.51	1-Tricosanol	4	807	823	6.18	05-01-3133	C_23_H_48_O	340
9	30.08	16467246	1.99	Eicosen-1-ol, cis-9	1	810	847	10.25	112248-30-3	C_20_H_40_O	296
10	30.7	56408242	7.01	17-Pentatriacontene	1	799	800	7.41	6971-40-0	C_35_H_70_	490
11	31.95	74961756	9.23	1-Tetracosanol	2	804	821	12.41	506-51-4	C_24_H_50_O	354
12	33.31	16764245	2.12	13-Docosen-1-ol, (Z)	1	798	820	9.17	629-98-1	C_22_H_44_O	324
13	33.17	68435403	8.53	1-Hentetracontanol	1	848	870	37.4	40710-42-7	C_41_H_84_O	592
14	35.01	229932016	28.21	1-Pentacosanol	1	812	823	31.85	26040-98-2	C_25_H_52_O	368
15	35.5	12157246	1.49	1,30-Triacontanediol	1	769	786	7.65	36645-68-8	C_30_H_62_O_2_	454
16	36.4	29211487	3.47	1-Heptacosane	3	781	825	10.3	593-49-7	C_27_H_56_	380
17	36.74	5914760	0.73	α-Tocopherol-β-D-mannoside	1	814	865	57.26	CID 597057	C_35_H_60_O_7_	592
18	37.95	54630744	6.4	13-Tetradecen-1-ol acetate	5	743	801	4.89	56221-91-1	C_16_H_30_O_2_	254
19	38.08	6914671	0.9	Campesterol	1	771	794	53.27	474-62-4	C_28_H_48_O	400
20	39.39	107745880	13.4	Stigmast-5-en-3-ol, (3β,24S)	1	848	855	45.49	83-47-6	C_29_H_50_O	414
21	39.62	5803953	0.71	Stigmastanol	1	720	732	58.69	19466-47-8	C_29_H_52_O	416
22	41.64	10789041	1.28	Stigmast-4-en-3-one	1	699	874	18.43	1058-61-3	C_29_H_48_O	412

#### Chloroform root extract

GC/MS chemometric profile of chloroform root extract showed the presence of 18 different chemotypes (Table 2, Fig. 2). Amongst these, stigmast-5-en-3-ol, (3β,24S) (24.30%), methyl tri-butyl ammonium chloride (14.64%), bis(2-ethylhexyl) phthalate (11.50%), 7,8-dimethylbenzocyclooctene (7.97%), ethyl linoleate (4.75%), 3-methoxy-5-methylphenol (4.16%), and hexadecanoic acid (4.13%) were found to constitute major amount while, campesterol (3.94%), 1-pentacosanol (3.82%), 17-pentariacontene (3.38%), benzene sulfonic acid, 4-amino-3-nitro (3.21%), orcinol (2.93%), benzenemethanol, 3-hydroxy, 5-methoxy (2.62%), 1-hentetracontanol (2.54%), 1-tetracosanol (1.86%), stigmast-4-en-3-one (1.82%); and α-tocopherol (1.31%), and eicosen-1-ol, cis-9 (1.13%) were found to be present in trace quantity.

**Table 2:** Phyto-chemotypes identified in the chloroform root extract of *R. imbricata* by GC/MS.

S. No.	Peak RT (min)	Peak area	Peak area %	Compound detected	Hit	SI	RSI	Prob	CAS No	Mol. Formula	Mol. Wt.
1	11.69	9872792	14.64	Methyl tri-butyl ammonium chloride	1	792	797	56.37	56375-79-2	C_13_H_30_ClN	235
2	13.75	2364627	4.16	3-Methoxy-5-methylphenol	1	808	858	67.78	3209-13-0	C_8_H_10_O_2_	138
3	15.14	1927265	2.93	1,3-Benzenediol, 5-methyl	1	708	865	22.77	504-15-4	C_7_H_8_O_2_	124
4	18.35	1520584	2.62	Benzenemethanol, 3-hydroxy-5-methoxy	1	811	860	85.91	30891-29-3	C_8_H_10_O_3_	154
5	20.55	5041042	7.97	7,8-Dimethylbenzocyclooctene	1	770	849	30.04	99027-76-6	C_14_H_14_	182
6	22.89	2762363	4.13	Hexadecanoic acid	1	749	813	60.75	57-10-3	C_16_H_32_O_2_	256
7	25.01	3042142	4.75	Ethyl linoleate	4	736	854	17.03	544-35-4	C_20_H_36_O_2_	308
8	28.8	2066650	3.21	Benzene sulfonic acid, 4-amino-3-nitro	4	582	652	2.29	616-84-2	C_6_H_6_N_2_O_5_S	218
9	29.11	7589941	11.5	Bis(2-ethylhexyl) phthalate	4	793	806	9.51	117-81-7	C_24_H_38_O_4_	390
10	30.12	670458	1.13	Eicosen-1-ol, cis-9	1	810	847	10.25	112248-30-3	C_20_H_40_O	296
11	30.74	2150315	3.38	17-Pentariacontene	1	799	800	7.41	6971-40-0	C_35_H_70_	490
12	31.98	1129772	1.86	1-Tetracosanol	2	804	821	12.41	506-51-4	C_24_H_50_O	354
13	33.23	1665168	2.54	1-Hentetracontanol	1	848	870	37.4	40710-42-7	C_41_H_84_O	592
14	34.99	2475797	3.82	1-Pentacosanol	8	616	763	0.8	26040-98-2	C_25_H_52_O	368
15	36.83	898525	1.31	α-Tocopherol	3	571	702	11.35	59-02-9	C_29_H_50_O_2_	430
16	38.17	2267712	3.94	Campesterol	1	682	779	20.92	474-62-4	C_28_H_48_O	400
17	39.51	16343303	24.29	Stigmast-5-en-3-ol, (3β,24S)	1	798	823	43.31	83-47-6	C_29_H_50_O	414
18	41.82	1236875	1.82	Stigmast-4-en-3-one	1	540	625	8.51	1058-61-3	C_29_H_48_O	412

#### Dichloroethane root extract

GC/MS chemometric profile of dichloroethane root extract illustrated the presence of 25 different chemotypes (Table 3, Fig. 3). Among these, camphor (17.78%), stigmast-5-en-3-ol, (3β,24S) (15.42%), ethyl linoleate (9.95%), 1,3-dimethoxybenzene (8.15%), hexadecanoic acid (6.55%), and thujone (4.73%) were present in major amount, whereas, benzene sulfonic acid, 4-amino-3-nitro (3.96%), campesterol (3.88%), methanol, (4-carboxymethoxy) benzoyl (3.27%), stigmast-4-en-3-one (2.84%), 1-hentetracontanol (2.74%), oleic acid (2.18%), bis(2-ethylhexyl) adipate (2.16%), bacteriochlorophyll-c-stearyl (2.11%), eucalyptol (1.95%), ethanone, 1-(2,6-dihydroxy-4-methoxyphenyl) (1.66%), 1-dotriacontane (1.58%), linalyl isovalerate (1.49%), 3-methoxy-5-methylphenol (1.47%), 1-chloro-2,4-dimethoxybenzene (1.44%), borneol (1.22%), 4-chlorothiophenol (1.02%), phenol, 2,4-bis(1,1-dimethylethyl) (0.94%), fenchyl alcohol (0.91%), and stigmast-3,5-dien-7-one (0.60%) were found to be present in trace.

**Table 3:** Phyto-chemotypes identified in the dichloroethane root extract of *R. imbricata* by GC/MS.

S. No.	Peak RT (min)	Peak area	Peak area %	Compound detected	Hit	SI	RSI	Prob	CAS No	Mol. Formula	Mol. Wt.
1	8.19	1187393	1.95	Eucalyptol	2	800	829	51.1	470-82-6	C_10_H_18_O	154
2	9.64	2910856	4.73	Thujone	1	835	845	23.35	546-80-5	C_10_H_16_O	152
3	10.44	11188301	0.85	Camphor	1	837	854	25.13	76-22-2	C_10_H_16_O	152
4	10.91	742204	1.22	Borneol	1	866	878	28.4	464-45-9	C_10_H_18_O	154
5	11.32	552901	0.91	β-fenchyl alcohol	1	765	836	10.05	470-08-6	C_10_H_18_O	154
6	13.06	631861	1.02	Benzenethiol, 4-chloro	1	599	670	34.81	106-54-7	C_6_H_5_ClS	144
7	13.66	898266	1.47	3-Methoxy-5-methylphenol	1	759	851	50.29	3209-13-0	C_8_H_10_O_2_	138
8	15.88	2126709	3.27	Methanol, (4-carboxymethoxy) benzoyl	1	694	746	16.43	80099-44-1	C_10_H_10_O_5_	210
9	16.5	571000	0.94	Phenol, 2,4-bis(1,1-dimethylethyl)	2	842	865	22.66	96-76-4	C_14_H_22_O	206
10	17.36	885243	1.44	1-Chloro-2,4-dimethoxybenzene	1	635	756	24.81	7051-13-0	C_8_H_9_ClO_2_	172
11	17.56	926151	1.49	Linalyl isovalerate	1	751	812	14.87	50649-12-2	C_15_H_26_O_2_	238
12	19.33	953645	1.58	1-Dotriacontane	1	787	803	45.91	544-85-4	C_32_H_66_	450
13	20.49	975679	1.66	Ethanone, 1-(2,6-dihydroxy-4-methoxyphenyl)	1	661	835	45.49	7507-89-3	C_9_H_10_O_4_	182
14	22.81	4154976	6.55	Hexadecanoic acid	1	801	855	70.5	57-10-3	C_16_H_32_O_2_	256
15	23.18	1089256	2.18	Oleic acid	1	772	798	49.32	112-80-1	C_18_H_34_O_2_	282
16	24.28	1364685	2.11	Bacteriochlorophyll-c-stearyl	1	755	767	13.97	CID5367801	C_52_H_72_MgN_4_O_4_	840
17	24.94	6245279	9.95	Ethyl linoleate	1	783	883	10.73	544-35-4	C_20_H_36_O_2_	308
18	27.61	1330635	2.16	Hexanedioic acid, bis(2-ethylhexyl) ester	1	695	773	29.62	103-23-1	C_22_H_42_O_4_	370
19	28.74	2480706	3.96	Benzene sulfonic acid, 4-amino-3-nitro	6	590	655	1.81	616-84-2	C_6_H_6_N_2_O_5_S	218
20	29.1	5070293	8.15	1,3-Dimethoxybenzene							
21	33.12	1747914	2.74	1-Hentetracontanol	4	675	828	4.63	40710-42-7	C_41_H_84_O	592
22	38.04	2440601	3.88	Campesterol	1	652	759	13.55	474-62-4	C_28_H_48_O	400
23	39.36	9725435	15.42	Stigmast-5-en-3-ol, (3β,24S)	1	814	853	63.99	83-47-6	C_29_H_50_O	414
24	40.9	366122	0.6	Stigmast-3,5-dien-7-one	1	512	760	71	2034-72-2	C_29_H_46_O	410
25	41.65	1869647	2.84	Stigmast-4-en-3-one	1	628	856	22.86	1058-61-3	C_29_H_48_O	412

#### Ethyl acetate root extract

Nineteen different chemotypes were identified in ethyl acetate extract (Table 4, Fig. 4). Amongst these 19 chemotypes, 1,3-dimethoxybenzene (27.61%), 1,3-benzenediol, 5-pentadecyl (16.90%), 3-methoxy-5-methylphenol (10.11%), 1,3-benzenediol, 5-methyl (8.40%), benzenemethanol, 3-hydroxy, 5-methoxy (5.75%), cholest-4-ene-3,6-dione (5.75%), and dodecanoic acid, 3-hydroxy (4.46%) were found to constitute major amount, whereas, 7,8-dimethylbenzocyclooctene (3.57%) 3,5-dimethoxyphenyl acetate (3.44%), α-D-glucopyranoside, O-α-D-glucopyranosyl-(1.fwdarw.3)-β-D-fructofuranosyl (2.95%), stigmast-5-en-3-ol, (3β,24S) (2.12%), eicosen-1-ol, cis-9 (2.12%), hexadecanoic acid (1.84%), oleic acid (1.34%), bacteriochlorophyll-c-stearyl (1.14%), phenol, 2,4-bis(1,1-dimethylethyl) (0.85%), 1-pentatricontene (0.72%), 1-dodecanol, 3,7,11-trimethyl (0.61%), and stigmast-4-en-3-one (0.32%) were found to be present in trace.

**Table 4:** Phyto-chemotypes identified in the ethyl acetate root extract of *R. imbricata* by GC/MS.

S. No.	Peak RT (min)	Peak area	Peak area %	Compound detected	Hit	SI	RSI	Prob	CAS No	Mol. Formula	Mol. Wt.
1	10.11	18961417	4.16	3-Methoxy-5-methylphenol	1	880	881	78.08	3209-13-0	C_8_H_10_O_2_	138
2	14.63	17158116	8.4	1,3-Benzenediol, 5-methyl	1	909	929	70.69	504-15-4	C_7_H_8_O_2_	124
3	16.5	1721777	0.85	Phenol, 2,4-bis(1,1-dimethylethyl)	1	860	887	30.17	96-76-4	C_14_H_22_O	206
4	17.78	1213849	0.61	1-Dodecanol, 3,7,11-trimethyl	1	696	721	3.99	6750-34-1	C_15_H_32_O	228
5	18.07	11340896	5.75	Benzenemethanol, 3-hydroxy-5-methoxy	1	866	868	75.89	30891-29-3	C_8_H_10_O_3_	154
6	19.54	7034263	3.44	Phenol, 3,5-dimethoxy acetate	7	636	829	4.41	23133-74-6	C_10_H_12_O_4_	196
7	20.4	7323033	3.57	7,8-Dimethylbenzocyclooctene	1	803	870	51.6	99027-76-6	C_14_H_14_	182
8	20.6	4129209	2.12	Eicosen-1-ol, cis-9	1	750	771	5.19	629-96-9	C_20_H_40_O	296
9	21.22	6328149	2.95	α-D-glucopyranoside, O-α-D-glucopyranosyl-(1.fwdarw.3)-β-D-fructofuranosyl	1	720	759	37.89	597-12-6	C_18_H_32_O_16_	504
10	22.8	3545343	1.84	Hexadecanoic Acid	1	795	837	71.82	57-10-3	C_16_H_32_O_2_	256
11	23.17	2659100	1.34	Oleic acid	1	779	794	11.53	112-80-1	C_18_H_34_O_2_	282
12	24.97	9179686	4.46	Dodecanoic acid, 3-hydroxy	1	674	706	35.46	1883-13-2	C_12_H_24_O_3_	216
13	25.52	2261098	1.14	Bacteriochlorophyll-c-stearyl	1	722	739	6.88	CID5367801	C_52_H_72_MgN_4_O_4_	840
14	27.68	1352685	0.72	17-Pentatriacontene	1	720	737	46.41	6971-40-0	C_35_H_70_	490
15	29.08	76996880	27.61	1,3-Dimethoxybenzene	2	712	767	11.52	151-10-0	C_8_H_10_O_2_	138
16	30.51	29595091	16.9	1,3-Benzenediol, 5-pentadecyl	1	664	797	23.45	3158-56-3	C_21_H_36_O_2_	320
17	32.06	11993388	5.75	Cholest-4-ene-3,6-dione	1	710	773	32.12	984-84-9	C_27_H_42_O_2_	398
18	39.36	4575754	2.12	Stigmast-5-en-3-ol, (3β,24S)	1	757	821	54.07	83-47-6	C_29_H_50_O	414
19	41.64	684162	0.32	Stigmast-4-en-3-one	1	508	755	16.81	1058-61-3	C_29_H_48_O	412

#### Methanol root extract

The methanol root extract revealed the presence of 18 different chemotypes (Table 5, Fig. 5). Among the identified chemotypes, stigmast-5-en-3-ol, (3β,24S) (21.91%), octadecane, 1-chloro (17.01%), ethanone, 1-(4-hydroxyphenyl) (11.07%), α-tocopherol (8.42%), ascaridole (5.92%), and campesterol (4.98%) were found to present in major amount, while, linolein, 2-mono (3.99%), hexadecanoic acid (3.67%), 1,3-dimethoxybenzene (3.57%), ethyl linoleate (3.35%), 1-dotriacontane (2.21%), linolein, 1-mono (1.74%), methyl palmitate (1.73%), stigmast-4-en-3-one (1.55%), 1-dodecane (0.66%), δ-tocopherol (0.56%), and 3-methoxy-5-methylphenol (0.43%) were found to be present in trace.

**Table 5:** Phyto-chemotypes identified in the methanol root extract of *R. imbricata* by GC/MS.

S. No.	Peak RT (min)	Peak area	Peak area %	Compound detected	Hit	SI	RSI	Prob	CAS No	Mol. Formula	Mol. Wt.
1	13.73	224745	0.43	3-Methoxy-5-methylphenol	1	730	810	72.09	3209-13-0	C_8_H_10_O_2_	138
2	15.78	5548712	11.07	Ethanone, 1-(4-hydroxyphenyl)	1	884	910	60.39	99-93-4	C_8_H_8_O_2_	136
3	17.87	345721	0.66	1-Dodecane	1	699	751	16.38	112-40-3	C_12_H_26_	170
4	19.33	1157289	2.21	1-Dotriacontane	1	777	785	14.73	544-85-4	C_32_H_66_	450
5	20.69	8405167	17.01	Octadecane, 1-chloro	1	734	738	22.3	386-33-2	C_18_H_37_Cl	288
6	22.32	905705	1.73	Hexadecanoic acid, methyl ester	1	793	866	61.21	112-39-0	C_17_H_34_O_2_	270
7	22.89	1901742	3.67	Hexadecanoic acid	4	660	762	11.87	57-10-3	C_16_H_32_O_2_	256
8	24.36	1592833	3.99	9,12-Octadecadienoic acid (Z,Z)-, 2-hydroxy-1-(hydroxymethyl)ethyl ester	5	807	833	6.93	3443-82-1	C_21_H_38_O_4_	354
9	24.44	821776	1.74	9,12,15-Octadecatrienoic acid, 2,3-dihydroxypropyl ester, (Z,Z,Z)	2	804	825	32.86	18465-99-1	C_21_H_36_O_4_	352
10	24.96	1495805	3.35	Ethyl linoleate	10	681	835	2.99	544-35-4	C_20_H_36_O_2_	308
11	29.06	1614826	3.57	1,3-Dimethoxybenzene	34	471	675	0.33	151-10-0	C_8_H_10_O_2_	138
12	30.91	3109377	5.92	Ascaridole	1	619	700	57.05	512-85-6	C_10_H_16_O_2_	168
13	31.92	3533697	7.23	Unknown	-	-	-	-	-	-	-
14	33.77	174824	0.56	δ-Tocopherol	1	667	763	89.78	119-13-1	C_27_H_46_O_2_	402
15	36.71	4419092	8.42	α-Tocopherol	1	731	853	46.36	59-02-9	C_29_H_50_O_2_	430
16	38.04	2615057	4.98	Campesterol	1	726	810	24.4	474-62-4	C_28_H_48_O	400
17	39.36	11245680	21.91	Stigmast-5-en-3-ol, (3β,24S)	1	813	850	67.6	83-47-6	C_29_H_50_O	414
18	41.38	902689	1.55	Stigmast-4-en-3-one	45	395	709	0.74	1058-61-3	C_29_H_48_O	412

#### 60% Ethanol root extract

GC/MS chemometric profile of 60% ethanol root extracts illustrated the presence of 12 different chemotypes (Table 6, Fig. 6). Amongst the identified chemotypes, dotriacontane (5.69%), and heptadecane, 9-hexyl (5.44%) were found to be present in major amount, whereas, bis(2-ethylhexyl) phthalate (3.58%), hexadecanoic acid, methyl ester (2.27%), and dibutyl phthalate (1.23%) were found to be present in trace.

**Table 6:** Phyto-chemotypes identified in the 60% ethanol root extract of *R. imbricata* by GC/MS.

S. No.	Peak RT (min)	Peak area	Peak area %	Compound detected	Hit	SI	RSI	Prob	CAS No	MF	MW
1	19.37	258681	5.69	1-Dotriacontane	1	769	805	39.82	544-85-4	C_32_H_66_	450
2	20.72	276232	5.44	Heptadecane, 9-hexyl	1	696	719	24.93	55124-79-3	C_23_H_48_	324
3	21.56	62567	1.23	Dibutyl phthalate	5	799	875	5.32	84-74-2	C_16_H_22_O_4_	278
4	22.36	115053	2.27	Hexadecanoic acid, methyl ester	1	624	700	20.11	112-39-0	C_17_H_34_O_2_	270
5	23.22	296422	5.84	Unknown	-	-	-	-	-	-	-
6	24.48	194215	3.83	Unknown	-	-	-	-	-	-	-
7	25.41	56168	1.11	Unknown	-	-	-	-	-	-	-
8	29.1	181582	3.58	Bis(2-ethylhexyl) phthalate	2	697	786	11.81	117-81-7	C_24_H_38_O_4_	390
9	34.21	644518	12.7	Unknown	-	-	-	-	-	-	-
10	39.51	1832781	35.53	Unknown	-	-	-	-	-	-	-
11	39.95	550771	10.86	Unknown	-	-	-	-	-	-	-
12	40.92	604576	11.92	Unknown	-	-	-	-	-	-	-

## Discussion

We have conducted the present investigation to identify the major volatile and semivolatile components in the root of *R. imbricata*. The presence of various bioactive compounds justifies the use of the plant by traditional practitioners of ‘Amchi’ system of medicine in trans-Himalayan Ladakh region. Also, extensive pharmacological studies were conducted by different researchers with the plant root extracts [6]–[23] and the results were very promising to justify the use of this plant as therapeutic agent.

However, the phytochemical profiling of the plant root still remains to be unexplored and to the best of our knowledge, this is the first ever study of its kind on the GC/MS chemometric profiling of the root extracts. In medicinal chemistry, it is very essential to ascertain the chemotyping of medicinal plant parts that are responsible for its numerous pharmacological properties and by this technique we may be able to scientifically determine and validate the traditional uses, pharmacological activities, and therapeutic potential of these plant parts. Profiling of metabolites in plant extracts permits the complete phenotyping of genetically or environmentally adapted plant systems and such investigations draw on simple extraction procedures that have been shown to be very robust and have permitted broad range of high-throughput applications in plant metabolomics. [25]–[27].

The major phytochemical groups in n-hexane, ethyl acetate, and 60% ethanol extracts were saturated alcohol (50%), phenols (40%), and alkanes (61%) respectively. On the other hand, phytosterols were the major group in chloroform (31%), dichloroethane (27%), and methanol (40%) extracts. The total of various volatile and semi volatile groups present in different root extracts of *R. imbricata* had the following distribution order: phytosterols (122%), alkanes (83%), phenols (69.46%), esters (48%), ethers (44%), fatty acid esters (43%), fatty acids (33%), terpenoids (18%), arenes (16%), alkyl ammonium halide salt (15%), alkenes (10%), sulfonic acid (8%), unsaturated alcohols (8%), organic acids (4%), saturated alcohols (4%), glycosides (3%), photosynthetic pigments (3%), steroidal glycoside (1%). The order of extraction capacities of different polarity solvents for phytosterols, phenols, fatty acids, alkanes, esters, fatty acid esters, ethers, unsaturated alcohols, arenes, terpenoids, alkenes, sulfonic acid, photosynthetic pigment, and saturated alcohols was as follows:

1. Phytosterols: methanol (40%), chloroform (31%), dichloroethane (27%), n-hexane (16%), ethyl acetate (8%)
2. Phenols: ethyl acetate (40%), methanol (13%), chloroform (10%), dichloroethane (6%), n-hexane (0.46%)
3. Fatty acids: dichloroethane (10%) = methanol (10%), ethyl acetate (8%), chloroform (4%), n-hexane (1%)
4. Alkane: 60% ethanol (61%), n-hexane (9%), chloroform (8%), dichloroethane (5%)
5. Esters: 60% ethanol (26%), chloroform (12%), n-hexane (8%), dichloroethane (2%)
6. Fatty acid esters: dichloroethane (15%), 60% ethanol (13%), methanol (6%), chloroform (5%), n-hexane (4%)
7. Ethers: ethyl acetate (28%), dichloroethane (12%), methanol (4%)
8. Unsaturated alcohols: n-hexane (4%), ethyl acetate (3%), chloroform (1%)
9. Arenes: chloroform (8%) = ethyl acetate (8%)
10. Terpenoids: dichloroethane (12%), methanol (6%)
11. Alkenes: n-hexane (7%), chloroform (3%)
12. Sulfonic acid: dichloroethane (5%), chloroform (3%)
13. Photosynthetic pigment: dichloroethane (2%), ethyl acetate (1%)
14. Saturated alcohols: methanol (3%), ethyl acetate (1%)

**Figure 7:**
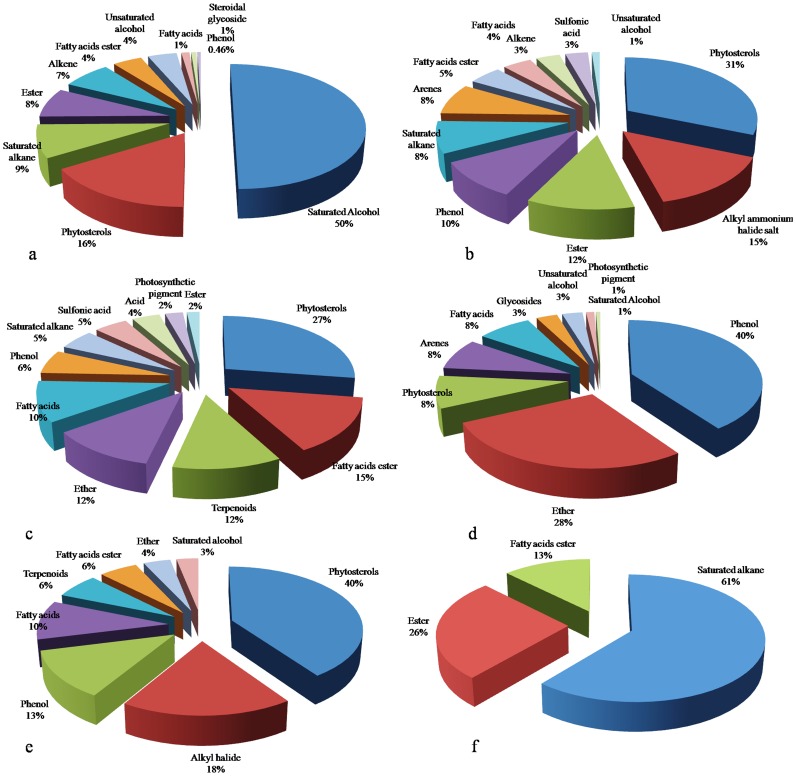
Estimation of major phytochemical groups in different root extracts of *R. imbricata*, a) n-hexane extract, b) chloroform extract, c) dichloroethane extract, d) ethyl acetate extract, e) methanol extract, f) 60% ethanol extract.

**Figure 8:**
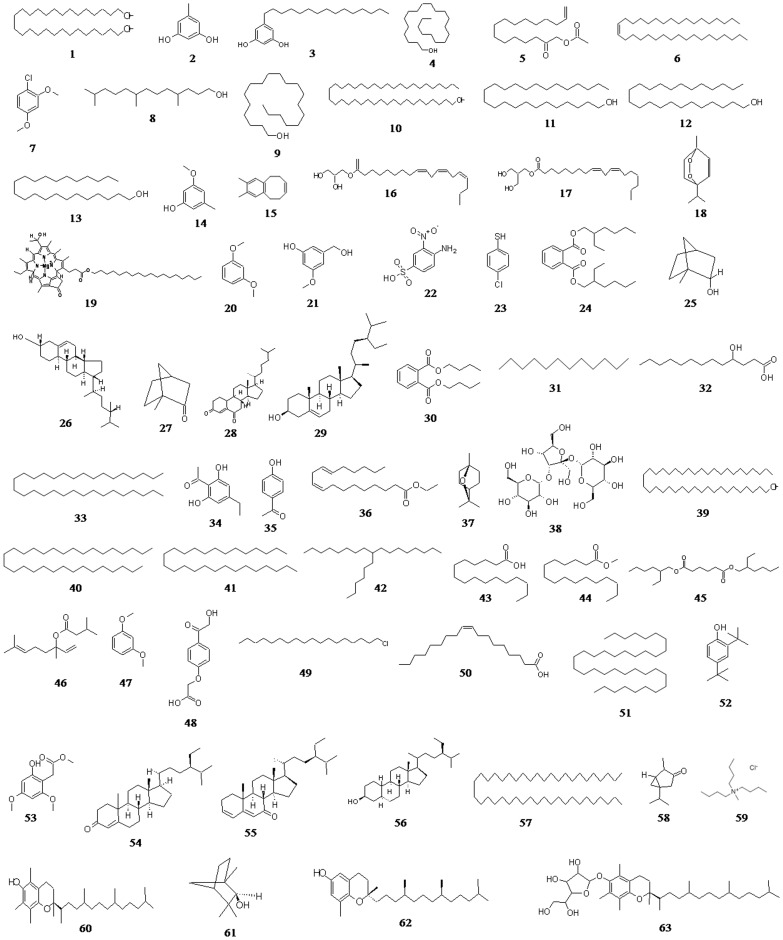
Phyto-chemotypes identified in different root extracts of *R. imbricata*. **1:** 1,30-triacontanediol; **2:** 1,3-benzenediol, 5-methyl; **3:** 1,3-benzenediol, 5-pentadecyl; **4:** 13-docosen-1-ol, (Z); **5:** 13-tetradecen-1-ol acetate; **6:** 17-pentatriacontene; **7:** 1-chloro-2,4-dimethoxybenzene; **8:** 1-dodecanol, 3,7,11-trimethyl; **9:** eicosen-1-ol, cis-9; **10:** 1-hentetracontanol; **11:** 1-pentacosanol; **12:** 1-tetracosanol; **13:** 1-tricosanol; **14:** 3-methoxy-5-methylphenol; **15:** 7,8-dimethylbenzocyclooctene; **16:** 9,12,15-octadecatrienoic acid, 2,3-dihydroxypropyl ester, (Z,Z,Z); **17:** 9,12-octadecadienoic acid (Z,Z)-, 2-hydroxy-1-(hydroxymethyl)ethyl ester; **18:** ascaridole; **19:** bacteriochlorophyll-c-stearyl; **20:** benzene, 1,3-dimethoxy; **21:** benzenemethanol, 3-hydroxy-5-methoxy; **22:** benzene sulfonic acid, 4-amino-3-nitro; **23:** benzenethiol, 4-chloro; **24:** bis(2-ethylhexyl) phthalate; **25:** borneol; **26:** campesterol; **27:** camphor; **28:** cholest-4-ene-3,6-dione; **29:** stigmast-5-en-3-ol, (3β,24S); **30:** di-butyl phthalate; **31:** 1-dodecane; **32:** dodecanoic acid, 3-hydroxy; **33:** 1-dotriacontane; **34:** ethanone, 1-(2,6-dihydroxy-4-methoxyphenyl); **35:** ethanone, 1-(4-hydroxyphenyl); **36:** ethyl linoleate; **37:** eucalyptol; **38:** α-D-glucopyranoside, O-α-D-glucopyranosyl-(1.fwdarw.3)-β-D-fructofuranosyl; **39:** 1-hentetracontanol; **40:** 1-hentriacontane; **41:** 1-heptacosane; **42:** heptadecane, 9-hexyl; **43:** hexadecanoic acid; **44:** hexadecanoic acid, methyl ester; **45:** hexanedioic acid, bis(2-ethylhexyl) ester; **46:** linalyl isovalerate; **47:** 1,3-dimethoxybenzene; **48:** methanol, (4-carboxymethoxy)benzoyl; **49:** octadecane, 1-chloro; **50:** oleic acid; **51:** 1-pentatriacontane; **52:** phenol, 2,4-bis(1,1-dimethylethyl); **53:** phenol, 3,5-dimethoxy acetate; **54:** stigmast-4-en-3-one; **55:** stigmast-3,5-dien-7-one; **56:** stigmastanol; **57:** 1-tetratetracontane; **58:** thujone; **59:** methyl tri-butyl ammonium chloride; **60:** α-tocopherol; **61:** β-fenchyl alcohol; **62:** δ-tocopherol; **63:** α-tocopherol-β-D-mannoside.

The steroidal glycoside, alkyl ammonium halide salt, organic acids, and glycoside were found only in n-hexane (1%), chloroform (15%), dichloroethane (4%), and ethyl acetate (3%), respectively. Eventually, in the present study we have found phytosterols, terpenoids, fatty acids, fatty acid esters, alkyl halides, phenols, alcohols, ethers, alkanes, and alkenes as the major group of phyto-chemotypes in the different root extracts of *R. imbricate* (Fig. 7, Table 7). All these compounds identified by GC/MS analysis (Fig. 8) were further investigated for their biological activities [28] and most of them were found to possess a diverse range of positive pharmacological actions (Table 8).

**Table 7:** Distribution of phyto-chemotypes in different root extracts of *R. imbricatea*.

Phyto-chemotypes	Root extracts					
	n-Hexane	Chloroform	Dichloroethane	Ethyl acetate	Methanol	60% Ethanol
1,30-Triacontanediol	√	–	–	–	–	–
1,3-Benzenediol, 5-methyl	–	√	–	√	–	–
1,3-Benzenediol, 5-pentadecyl	–	–	–	√	–	–
13-Docosen-1-ol, (Z)	√	–	–	–	–	–
13-Tetradecen-1-ol acetate	√	–	–	–	–	–
17-Pentatriacontene	√	√	–	√	–	–
1-Chloro-2,4-dimethoxybenzene	–	–	√	–	–	–
1-Dodecanol, 3,7,11-trimethyl	–	–	–	√	–	–
Eicosen-1-ol, cis-9	–	–	–	√	–	–
1-Hentetracontanol	√	–	√	–	–	–
1-Pentacosanol	√	√	–	–	–	–
1-Tetracosanol	√	√	–	–	–	–
1-Tricosanol	√	–	–	–	–	–
3-Methoxy-5-methylphenol	√	√	√	√	√	–
7,8-Dimethylbenzocyclooctene	–	√	–	√	–	–
9,12,15-Octadecatrienoic acid, 2,3-dihydroxypropyl ester, (Z,Z,Z)	–	–	–	–	√	–
9,12-Octadecadienoic acid (Z,Z)-, 2-hydroxy-1-(hydroxymethyl)ethyl ester	–	–	–	–	√	–
Ascaridole	–	–	–	–	√	–
Bacteriochlorophyll-c-stearyl	–	–	√	√	–	–
Benzene, 1,3-dimethoxy	–	–	–	√	√	–
Benzenemethanol, 3-hydroxy-5-methoxy	–	√	–	√	–	–
Benzene sulfonic acid, 4-amino-3-nitro	–	√	√	–	–	–
Benzenethiol, 4-chloro	–	–	√	–	–	–
Bis(2-ethylhexyl) phthalate	√	√	–	–	–	√
Borneol	–	–	√	–	–	–
Campesterol	√	√	√	–	√	–
Camphor	–	–	√	–	–	–
Cholest-4-ene-3,6-dione	–	–	–	√	–	–
Stigmast-5-en-3-ol, (3β,24S)	√	√	√	√	√	–
Di-butyl phthalate	–	–	–	–	–	√
1-Dodecane	–	–	–	–	√	–
Dodecanoic acid, 3-hydroxy	–	–	–	√	–	–
1-Dotriacontane	–	–	√	–	√	√
Ethanone, 1-(2,6-dihydroxy-4-methoxyphenyl)	–	–	√	–	–	–
Ethanone, 1-(4-hydroxyphenyl)	–	–	–	–	√	–
Ethyl linoleate	√	√	√	–	√	–
Eucalyptol	–	–	√	–	–	–
α-D-Glucopyranoside, O-α-D-glucopyranosyl-(1.fwdarw.3)-β-D-fructofuranosyl	–	–	–	√	–	–
1-Hentetracontanol	–	√	–	–	–	–
1-Hentriacontane	√	–	–	–	–	–
1-Heptacosane	√	–	–	–	–	–
Heptadecane, 9-hexyl	–	–	–	–	–	√
Hexadecanoic acid	√	√	√	√	√	–
Hexadecanoic acid, methyl ester	–	–	–	–	√	√
Hexanedioic acid, bis(2-ethylhexyl) ester	–	–	√	–	–	–
Linalyl isovalerate	–	–	√	–	–	–
1,3-Dimethoxybenzene	–	–	√	–	–	–
Methanol, (4-carboxymethoxy)benzoyl	–	–	√	–	–	–
Octadecane, 1-chloro	–	–	–	–	√	–
Oleic acid	–	–	√	√	–	–
1-Pentatriacontane	√	–	–	–	–	–
Phenol, 2,4-bis(1,1-dimethylethyl)	–	–	√	√	–	–
Phenol, 3,5-dimethoxy, acetate	–	–	–	√	–	–
Stigmast-4-en-3-one	√	√	√	√	√	–
Stigmast-3,5-dien-7-one	–	–	√	–	–	–
Stigmastanol	√	–	–	–	–	–
1-Tetratetracontane	√	–	–	–	–	–
Thujone	–	–	√	–	–	–
Methyl tri-butyl ammonium chloride	–	√	–	–	–	–
α-Tocopherol	√	√	–	–	√	–
β-Fenchyl alcohol	–	–	√	–	–	–
δ-Tocopherol	–	–	–	–	√	–
α-Tocopherol-β-D-mannoside	√	–	–	–	–	–

√ Present; – Absent.

**Table 8:** Biological activities of active principles present in different root extracts of *R. imbricate*.

Phyto-chemotypes	Biological activity
Eicosen-1-ol, cis-9	Antimalarial, antifungal, antioxidant
1-Tricosanol	Antibacterial, antifungal
9,12,15-Octadecatrienoic acid, 2,3-dihydroxypropyl ester, (Z,Z,Z)	5-Alpha-reductase inhibitor, antiMS, antiacne, antialopecic, antianaphylactic, antiandrogenic, antiarteriosclerotic, antiarthritc, anticoronary, antieczemic, antifibrinolytic, antigranular, antihistaminic, antiinflammatory, antileukotriene-D4, antimenorrhagic, antiprostatitic, cancer-preventive, carcinogenic, comedolytic, hepatoprotective, hypocholesterolemic, immunomodulator, insectifuge, metastatic, nematicide, propecic
9,12-Octadecadienoic acid (Z,Z)-, 2-hydroxy-1-(hydroxymethyl)ethyl ester	Antiinflammatory, hypocholesterolemic, cancer preventive, hepatoprotective, nematicide, insectifuge, antihistaminic, antieczemic, antiacne, 5-alpha reductase inhibitor antiandrogenic, antiarthritic, anticoronary, insectifuge
Ascaridole	Analgesic, ancylostomicide, anthelmintic, antiflatulent, antimalarial, carcinogenic, carminative, fungicide, nematicide, pesticide, plasmodicide, sedative, transdermal, trypanocide, vermifuge
Borneol	(-)-Chronotropic, (-)-inotropic, allelochemic, analgesic, antiacetylcholine, antibacterial, antibronchitic, antiescherichic, antifeedant, antiinflammatory, antiotitic, antipyretic, antisalmonella, antispasmodic, antistaphylococcic, antiyeast, CNS-stimulant, CNS-toxic, candidicide, choleretic, flavor; fungicide, hepatoprotective, herbicide, herbicide, inhalant, insect-repellent, insectifuge, irritant, myorelaxant, nematicide, perfumery, pesticide, sedative, tranquilizer
Campesterol	Antioxidant, hypocholesterolemic
Camphor	Allelopathic, analgesica, anesthetic, antiacne, antidiarrheic, antidysenteric, antiemetic, antifeedant, antifibrositic, antineuralgic, antipruritic, antiseptic, antispasmodic, CNS-stimulant, cancer preventive, carminative, convulsant, cosmetic, counterirritant, decongestant, deliriant, ecbolic, emetic, epileptigenic, expectorant, fungicide, herbicide, insect-repellent, insectifuge, irritant, nematicide, occuloirritant, P450-2B1-inhibitor, pesticide, respirainhibitor, respirastimulant, rubefacient, stimulant, transdermal, verrucolytic, vibriocide
Stigmast-5-en-3-ol, (3β,24S)	Androgenic, angiogenic, anorexic, antiadenomic, antiandrogenic, antibacterial, anticancer (breast), anticancer (cervix), anticancer (lung), antiedemic, antiestrogenic, antifeedant, antifertility, antigonadotrophic, antihyperlipoproteinaemic, antiinflammatory, antileukemic, antilymphomic, antimutagenic, antiophidic, antioxidant, antiprogestational, antiprostaglandin, antiprostatadenomic, antiprostatitic, antipyretic, antitumor (breast), antitumor (cervix), antitumor (lung), antiviral, apoptotic, artemicide, cancer-preventive, candidicide, caspase-8-inducer, estrogenic, febrifuge, gonadotrophic, hepatoprotective, hypocholesterolemic, hypoglycemic, hypolipidemic, pesticide, spermicide, ubiquiot, ulcerogenic
Di-butyl phthalate	Antimicrobial, Antifouling
Dodecanoic acid, 3-hydroxy	Flavor
Eucalyptol	Anesthetic, anthelmintic, antibacterial, antihalitosic, antiseptic, antitussive, decongestant, expectorant, hypotensive, insectifuge, irritant, pesticide, vermicide
α-D-Glucopyranoside, O-α-D-glucopyranosyl-(1.fwdarw.3)-β-D-fructofuranosyl	Preservative
Hexadecanoic acid	Antioxidant, hypocholesterolemic, nematicide, pesticide, lubricant, antiandrogenic, flavor, hemolytic 5-alpha reductase inhibitor
Hexadecanoic acid, methyl ester	Antioxidant, nematicide, pesticide, lubricant, antiandrogenic, flavor, hemolytic 5-alpha reductase inhibitor, hypocholesterolemic
Linalyl isovalerate	Fragrance
Oleic acid	5-Alpha-reductase-inhibitor, allergenic, anemiagenic, antialopecic, antiandrogenic, antiinflammatory, antileukotriene-D4; cancer-preventive, choleretic, dermatitigenic, flavor, hypocholesterolemic, insectifuge, irritant, percutaneostimulant, perfumery, propecic
1-Pentatriacontane	Herbistat
Stigmast-4-en-3-one	Antiprostatitic
Stigmast-3,5-dien-7-one	Antifertility
Thujone	Abortifacient, anthelmintic, antibacterial, antiseptic, antispasmodic, cerebrodepressant, convulsant, counterirritant, emmenagogue, epileptigenic, hallucinogenic, herbicide, neurotoxic, perfumery, pesticide, respirainhibitor, toxic
β-Fenchyl alcohol	Antimicrobial, antioxidant, flavor
δ-Tocopherol	5-HETE-inhibitor, allergenic, analgesic, antiMD, antiMS, antiPMS, antiaggregant, antiaging, antialzheimeran, antianginal, antiarteriosclerotic, antiarthritic, antiatherosclerotic, antibronchitic, anticancer (breast), anticariogenic, anticataract, antichorea, antichoreic, anticonvulsant, anticoronary, antidecubitic, antidementia, antidermatitic, antidiabetic, antidysmenorrheic, antiepitheleomic, antifibrositic, antiglycosation, antiherpetic, antiinfertility, antiinflammatory, antiischemic, antileukemic, antileukotriene, antilithic, antilupus, antimaculitic, antimastalgic, antimelanomic, antimyoclonic, antineuritic, antineuropathic, antinitrosaminic, antiophthalmic, antiosteoarthritic, antioxidant, antiparkinsonian, antiproliferant, antiradicular, antiretinopathic, antirheumatic, antisenility, antisickling, antispasmodic, antisterility, antistroke, antisunburn, antisyndrome-X, antithalassemic, antithrombotic, antithromboxane-B2, antitoxemic, antitumor; antitumor (breast), antitumor (colorectal), antitumor (prostate), antitumor (stomach), antiulcerogenic, apoptotic, calcium-antagonist, cancer-preventive, cardioprotective, cerebroprotective, circulatory-stimulant, circulotonic, hepatoprotective, hypocholesterolemic, hypoglycemic, immunomodulator, immunostimulant, insulin-sparing, lipoxygenase-inhibitor, NO-inhibitor, ornithine-decarboxylase-inhibitor, P21-inducer, phospholipase-A2-inhibitor, protein-kinase-C-inhibitor, vasodilator

Most of the pharmacological studies were conducted with the aqueous, ethanol, and hydro-alcoholic root extracts of this plant and it was found to have numerous biological activities such as anti-stress, adaptogenic, anti-hypoxic, immune-stimulatory, anti-cancer, cytoprotective, radioprotective, anti-hemolytic, anti-inflammatory, and wound healing potential [6]–[23]. Our investigations conclude that the compounds present in the ethanol and water extracts have the potential to perform these functions. Though, the root extracts of the plant obtained by polar solvent extraction have been investigated for their pharmacological actions in considerable detail, non polar root extracts were not studied till date. Hence, our primary objective in the present work was to find the bioactive constituents present in the non polar extraction of root of this herb. These findings will definitely usher in new directions in pharmacological and therapeutic investigations with the root extracts obtained from non polar solvent extraction such as n-hexane, chloroform, dichloroethane, and ethyl acetate.

## Conclusion

In the present study, sixty three phyto-chemotypes have been identified from n-hexane, chloroform, dichloroethane, ethyl acetate, methanol, and 60% ethanol root extracts of *R. imbricata* by GC/MS analysis. It showed the existence of various bioactive principles that confirm the application of *R. imbricata* for various ailments in traditional system of medicine. However, isolation of individual phyto-chemotypes and subjecting them to biological activity will definitely give fruitful results to find a novel drug. It could be concluded that *R. imbricata* contains various bioactive phyto-chemotypes having phyto-pharmaceutical importance. However, further studies will need to be undertaken to ascertain its bioactivity, toxicity profile, effect on the ecosystem, and agricultural products.
